# Identification of a novel linear B-cell epitope in nonstructural protein 11 of porcine reproductive and respiratory syndrome virus that are conserved in both genotypes

**DOI:** 10.1371/journal.pone.0188946

**Published:** 2017-11-29

**Authors:** Nan Jiang, Huan Jin, Yi Li, Xinna Ge, Jun Han, Xin Guo, Lei Zhou, Hanchun Yang

**Affiliations:** 1 Key Laboratory of Animal Epidemiology of Ministry of Agriculture, College of Veterinary Medicine, China Agricultural University, Beijing, People’s Republic of China; 2 State Key Laboratory of Agrobiotechnology, China Agricultural University, Beijing, People’s Republic of China; Sun Yat-Sen University, CHINA

## Abstract

Porcine reproductive and respiratory syndrome virus (PRRSV) is one of the most economically important pathogens, that hinder the development of global pork industry. Its nonstructural protein 11 (nsp11), with the nidoviral uridylate-specific endoribonuclease (NendoU) domain, is essential for PRRSV genome replication and it also contributes to host innate immunity suppression. However, the immunogenicity and immune structure of PRRSV nsp11 have not been well investigated yet. In this study, a monoclonal antibody (mAb), designated 3F9, that against nsp11 was generated. Subsequently, a series of partially overlapped fragments, covered the nsp11_40-223aa_, were expressed to test the reactivity with mAb 3F9, and the ^111^DCREY^115^ was found to be the core unit of the B-cell epitope recognized by mAb 3F9. Further investigation indicated that both genotype 1 and genotype 2 PRRSV can be recognized by mAb 3F9, due to the ^111^DCREY^115^ is conserved in both genotype virus. Meanwhile, this epitope, localized at the surface of nsp11 in 3D structure, is confirmed to be able to induce humoral immune response in PRRSV infected pigs. These findings do not only provide an mAb tool to further investigate the function of nsp11, they also indicate the diagnostic potential for this epitope.

## Introduction

Porcine reproductive and respiratory syndrome (PRRS) is characterized as widespread reproductive failures in pregnant sow, including mummified, stillborn and aborted fetuses, as well as respiratory distress in all age pigs. Since it first emerged in the United States in late 1980s, as a “mystery” disease, PRRS had spread to most pig raising area of the world, which badly hinders the development of global pork industry [1–4]. The causative agent, porcine reproductive and respiratory syndrome virus (PRRSV), is classified in the order *Nidovirales*, family *Arteriviridae* and genus *Arterivirus*, with the prototype virus Equine Arterivirus (EAV) and two other members, lactate dehydrogenase elevating virus (LDV) and simian haemorrhagic fever virus (HSFV), with single-strand positive-sense RNA as genome and envelop at the outside of the virus [5]. Two major identified genotypes of PRRSV, initially circling in the Europe (genotype 1) and North American (genotype 2), were found to surprisingly genetically divergent, which have approximately ~40% nucleotide sequence difference, however these two genotypes present similar genomic organization, clinical symptoms, and temporal emergence [6]. Bioinformatics analysis indicated that these two types virus origin from the same ancestor, and processing a period independent evolution before the outbreak on the two continents [7]. Recently many countries have both genotypes’ strains emerged in the field, which increases the complexity of PRRSV diagnosis and prevention [8–10].

PRRSV genome is approximately 15kb in length with 12 overlapping open reading frames (ORFs) at least [11]. ORF1a and ORF1b occupied more than 70% of genome at the 5’ end, encode the replication-related pp1a and pp1ab, together with two ribosomal frame-shift (RFS) products pp1a-nsp2N, pp1a-nsp2TF, which are autoproteolytically cleaved into at least 16 nonstructural proteins (nsps) associated with virus replication and transcription [5, 12]. Among them, three important virus proteases, nsp1 (papain-like cysteine protease), nsp2 (chymotrypsin-like cysteine protease) and nsp4 (3C-like serine protease) in pp1a are associated with PRRSV nsp cleavage. And four crucial enzymes for PRRSV RNA synthesis, including nsp9 (RNA-dependent RNA polymerase, RdRp), nsp10 (RNA helicase), nsp11 (endoribonuclease) and nsp12 (functions unclear) are encoded by ORF1b [13]. The viral structural proteins encoded by ORFs 2 to 7, which involve in receptor binding, virus entry and neutralizing antibody elicitation etc., are individually expressed by a set of subgenomic RNAs (sgRNA) [14–16].

Nsp11 protein contains a highly conserved nidoviral uridylate-specific endoribonuclease (NendoU) domain unique for viruses in the order *Nidovirales* [17]. This domain resides in the C-terminal region of nsp11, showing a distant relationship with the XendoU family from Xenopus laevis [18–20]. The newly identified crystal structure suggests that the PRRSV nsp11 exists as an asymmetric dimmer [21], which differs from the hexametric structure of coronaviruses nsp15 [22, 23]. Based on previous mutation analysis study on EAV, the NendoU should be required for genome replication and particularly on sgRNA synthesis [18]. As well, nsp11 plays an important role associated with the suppression of type I IFN induction [24], the RNAi innate immune response [25] and the NLR family pyrin domain-containing 3 (NLRP3)-mediated productions of IL-1β [26]. Together with nsp1β, nsp11 is also responsible for different expression level of TNF-α, induced by PRRSV strains with different pathogenicity [27]. However, the characterization of nsp11 immunogenicity remains unclear. Whether the nsp11 could induce humoral immune response in PRRSV infected pigs, or if there is any B-cell epitope on nsp11, are worth to investigate.

In this study, a B-cell epitope in nsp11 protein was identified by using nsp11 specific mAb to react with a series of truncated nsp11 protein in western blotting (WB) test. This novel epitope was found to be conserved in both genotype strains, but not among other Arterivirus. The localization of this epitope on the surface of nsp11 makes it easy to induce immune responds during PRRSV infection. Therefore, B-cell epitope identification and characterizing the immunogenicity of PRRSV nsp11 does not only extend our understanding of the structure-function relationships and nonstructural protein induced immune response, it also indicates the potential possibility of applying nsp11 specific mAb for viral-host protein interaction research and diagnostic testing.

## Materials and methods

### Ethics statement

In this study, the animal trials were performed according to the Chinese Regulations of Laboratory Animals, Guidelines for the Care of Laboratory Animals (Ministry of Science and Technology of People’s Republic of China) and Laboratory Animal-Requirements of Environment and Housing Facilities (GB14925-2010, National Laboratory Animal Standardization Technical Committee). The Laboratory Animal Ethical Committee of China Agricultural University has approved this research protocol, with the issued license number as CAU20151120-1.

### Virus, plasmids, cells and serum samples

PRRSV genotype 1 strain GZ11-G1 and genotype 2 strain JXwn06, with GeneBank associate No. KF001144 and EF641008 respectively, were used for cell inoculation in IFA and WB tests [8, 28]. PRRSV full-length infectious clone plasmid pWSK-JXwn as well as two expression vector pET28a (Merck Millipore) and PEGFP-N1 (Clontech) were previously constructed or purchased in the lab [28]. MARC-145, HEK 293 and myeloma SP2/0 cells were cultured in Gibco Dulbecco’s modified Eagle medium (DMEM) (Invitrogen), which were supplemented with 10% fetal bovine serum (FBS) (HyClone), at 37°C under a humid 5% CO_2_ atmosphere. Pig serum samples collected from PRRSV inoculation trial or from pig farms, were confirmed to be PRRSV positive or negative by using IDEXX HerdChek PRRS X3 ELISA kit and the serum sample number for each group was 20.

### Expression and purification of recombinant protein

Truncated fragment covering 118–669 nt of nsp11 was amplified from the PRRSV full-length infectious clone plasmid pWSK-JXwn as a template with the primers nsp11-F/R listed in the Table 1, followed by cloning into the vector pET28a, using the the enzyme sites Bam HI and Sal I. The constructed His-fused nsp11 expressing plasmid pET28a-nsp11 was validated by DNA sequencing.

**Table 1:** Sequences of primers used in this study.

Name	Sequence (5’-3’)*
nsp11-F	GC GGATCC ACCCAGAACAATGAAAGGTGG
nsp11-R	GG GTCGAC TTCAAGTTGAAAATAGGCCGT
nsp11-T1 F	GCCTCGAGATGACCCAGAACAATGAAAG
nsp11-T1 R	GCGGATCCCGTTTTGTGAGATAGTATG
nsp11-T2 F	GCCTCGAGATGTCATACTATCTCACAAAATTTG
nsp11-T2 R	GCGGATCCCGGCCGATGAAGGCATG
nsp11-T3 F	GCCTCGAGATGCCACATGCCTTCATCG
nsp11-T3 R	GCGGATCCCGGTACACATCCGTCAATG
nsp11-T4 F	GCCTCGAGATGACATTGACGGATGTG
nsp11-T4 R	GCGGATCCCGTTCAAGTTGAAAATAGG
nsp11-T2-1 F	GCCTCGAGATGTCATACTATCTCACAAAATTTGTTAGAGGCGCCACCATGGTGAGCAAGGGC
nsp11-T2-1 R	GGCGGCCGCTTTACTTGTACAG
nsp11-T2-2 F	GCCTCGAGATGAAATTTGTTAGAGGCGAGGCTCAAGTGCTTGCCACCATGGTGAGCAAGGGC
nsp11-T2-2 R	GGCGGCCGCTTTACTTGTACAG
nsp11-T2-3 F	GCCTCGAGATGGAGGCTCAAGTGCTTCCGGAGACAGTCTTCGCCACCATGGTGAGCAAGGGC
nsp11-T2-3 R	GGCGGCCGCTTTACTTGTACAG
nsp11-T2-4 F	GCCTCGAGATGCCGGAGACAGTCTTCAGCACCGGCCGAATTGCCACCATGGTGAGCAAGGGC
nsp11-T2-4 R	GGCGGCCGCTTTACTTGTACAG
nsp11-T2-5 F	GCCTCGAGATGAGCACCGGCCGAATTGAGGTAGATTGTCGAGCCACCATGGTGAGCAAGGGC
nsp11-T2-5 R	GGCGGCCGCTTTACTTGTACAG
nsp11-T2-6 F	GCCTCGAGATGGAGGTAGATTGTCGAGAGTATCTTGATGATGCCACCATGGTGAGCAAGGGC
nsp11-T2-6 R	GGCGGCCGCTTTACTTGTACAG
nsp11-T2-7 F	GCCTCGAGATGGAGTATCTTGATGATCGGGAGCGAGAAGTTGCCACCATGGTGAGCAAGGGC
nsp11-T2-7 R	GGCGGCCGCTTTACTTGTACAG
nsp11-T2-8 F	GCCTCGAGATGCGGGAGCGAGAAGTTGCTGAGTCCCTCCCAGCCACCATGGTGAGCAAGGGC
nsp11-T2-8 R	GGCGGCCGCTTTACTTGTACAG
nsp11-T2-9 F	GCCTCGAGATGGCTGAGTCCCTCCCACATGCCTTCATCGGCGCCACCATGGTGAGCAAGGGC
nsp11-T2-9 R	GGCGGCCGCTTTACTTGTACAG
nsp11-T2-6-1 F	GCCTCGAGATGGAGGTAGATTGTCGAGAGGCCACCATGGTGAGCAAGGGC
nsp11-T2-6-1 R	GGCGGCCGCTTTACTTGTACAG
nsp11-T2-6-2 F	GCCTCGAGATGGAGGTAGATTGTCGAGAGTATGCCACCATGGTGAGCAAGGGC
nsp11-T2-6-2 R	GGCGGCCGCTTTACTTGTACAG
nsp11-T2-6-3 F	GCCTCGAGATGGAGGTAGATTGTCGAGAGTATCTTGCCACCATGGTGAGCAAGGGC
nsp11-T2-6-3 R	GGCGGCCGCTTTACTTGTACAG
nsp11-T2-6-4 F	GCCTCGAGATGGAGGTAGATTGTCGAGAGTATCTTGATGCCACCATGGTGAGCAAGGGC
nsp11-T2-6-4 R	GGCGGCCGCTTTACTTGTACAG
nsp11-T2-6-5 F	GCCTCGAGATGGTAGATTGTCGAGAGTATCTTGATGATGCCACCATGGTGAGCAAGGGC
nsp11-T2-6-5 R	GGCGGCCGCTTTACTTGTACAG
nsp11-T2-6-6 F	GCCTCGAGATGGATTGTCGAGAGTATCTTGATGATGCCACCATGGTGAGCAAGGGC
nsp11-T2-6-6 R	GGCGGCCGCTTTACTTGTACAG
nsp11-T2-6-7 F	GCCTCGAGATGTGTCGAGAGTATCTTGATGATGCCACCATGGTGAGCAAGGGC
nsp11-T2-6-7 R	GGCGGCCGCTTTACTTGTACAG
nsp11-T2-6-8 F	GCCTCGAGATGCGAGAGTATCTTGATGATGCCACCATGGTGAGCAAGGGC
nsp11-T2-6-8 R	GGCGGCCGCTTTACTTGTACAG

* Underlines indicate the enzyme sites used for cloning.

The plasmid pET28a-nsp11 was transformed into *Escherichia* coli BL21 (DE3) competent cells (Transgens) for protein expression. Cultures in logarithmic phase (at OD_600_ of ∼0.6–0.7) were induced at 37°C for 4 h with 1.0 mM isopropy-l-β-D-galactopyranoside (IPTG) (Sigma-Aldrich), followed by centrifugation for harvest. The pellets were washed twice by using 1x phosphate-buffered saline (PBS) and then re-suspended with 25ul PBS. Then bacterial cells were lysed by sonication, and the soluble and insoluble fractions of His-nsp11_40-223aa_ were divided by centrifugation at 12,000 rpm, 4°C for 10 min. Loading buffer was added into the samples which were then boiled for 5min before analysis on sodium dodecyl-sulfate polyacrylamide gel (SDS-PAGE). The Ni-Agarose His Purification Kit (CWB IOTECH) was used to purify the recombinant His-nsp11_40-223aa_ protein from thalluses part, according to the manufacturer’s instructions. The purity and concentration of purified nsp11_40-223aa_ protein were analyzed by SDS-PAGE, stained with Coomassie Blue.

### Preparation of monoclonal antibodies (mAbs) against nsp11 protein

The PRRSV nsp11 specific mAbs were generated according to established protocols with a few modifications [29]. Briefly, 6-weeks old BALB /c mice were primed subcutaneously with 100 ug of recombinant nsp11_40-223aa_ protein emulsified with Freund’s complete adjuvant (Sigma-Aldrich) in the equal volume. The booster vaccinations were carried out twice via peritoneum at 3 and 6 weeks after the primary immunization, using recombinant nsp11_40-223aa_ with equal volume of Freund′s incomplete adjuvant (Sigma-Aldrich). Three days after the final intraperitoneal injection booster with nsp11_40-223aa_ protein only, the mice were euthanized by cervical dislocation and the spleen cells were fused with SP2/0 myeloma cells using polyethylene glycol (PEG 1450) (Sigma-Aldrich), which were cultured in DMEM containing hypoxanthine-aminopterin-thymidine (HAT) (Sigma-Aldrich) and 20% FBS. The supernatant of the hybridomas was screened for secreting nsp11-specific antibodies with indirect enzyme-linked immunosorbent assay (ELISA) (protocols list below). Positive hybridomas were subcloned three rounds by limiting dilution, before using to produce ascites fluid by intraperitoneal injection of 5 × 10^5^ hybridoma cells into female BALB/c mice treated with 0.5 ml/mouse 2,6,10,14-tetramethylpentadecane (Pristane) (Sigma-Aldrich). The subclass specificity of mAbs was identified by the IsoQuickTM Strips and Kits for mouse monoclonal antibody isotyping (Roche Diagnostic Corporation).

### Indirect ELISA

Nsp11-ELISA was performed to screen antibodies against the nsp11 protein in the sera from immunized mice or supernatant of hybridoma culture. The protocol including concentration of coating antigens, sera dilution, coating time and condition was set up according to our previous report [30]. In brief, 96-well plate (Costar, Corning Incorporated) coated overnight with the purified His-nsp11_40-223aa_ protein (0.4ug/well) at 4°C, were blocked for 2 h with 5% (wt/vol) skim milk in PBS at 37°C followed by three times wash with PBST (containing 0.05% of Tween 20). The plates were then incubated with 100 uL diluted nsp11 mouse antisera or supernatants of hybridoma at 37°C for 1 h. After incubation and three times washing by PBST, the bound antibodies were detected with HRP-conjugated goat anti-mouse IgG (1: 5000 dilutions in PBST) (Sigma–Aldrich). After another 1 h inoculation and three times washing, the reaction was quantified by detecting the absorbance at 450 nm using 3, 3′, 5, 5′-Tetramethylbenzidine (TMB) (Sigma–Aldrich) as a substrate. Sample was considered as positive when the OD value was above the cutoff point the mean OD plus 3 S.D. of the negative sera (n = 8). The IDEXX HerdChek PRRS X3 ELISA kit confirmed PRRSV positive or negative sera, either from PRRSV inoculated SPF animals in previous study or collected from pig farms, were also submitted to serologically test for nsp11 specific antibodies, by using HRP-conjugated goat anti-pig IgG as secondary antibody.

For peptide indirect ELISA, the synthetic peptides (GenScript) for candidate epitope with the amino acid sequence of DCREY were cross-linked to 96-well Maxisorp plates, with the peptide amount of 5 ug/well (Nunc, Thermo Scientific), coated with 50 g/ml poly-L-lysine using 0.1% glutaraldehyde in PBS. Reactive aldehyde sites were blocked with 1 mol/L glycine at room temperature for 1 h, and plates were further blocked with 5% skim milk at 37°C for 2 h. The screened PRRSV positive or negative serum samples above, were diluted with blocking buffer (1:10) to detect their reactivity with coated peptides by an HRP-3,3 =, 5,5 = -tetramethylbenzidine (TMB) ELISA as described above, with an HRP-conjugated goat anti-pig IgG. Similarly, the hybridoma supernatant of screened monoclonal antibody and HRP-conjugated goat anti-mouse lgG were also used to test the reaction with coated peptides.

### Immunofluorescence assay

MARC-145 cells seeded into the 96-well micro-plate for monolayers, were inoculated with genotype 2 PRRSV strain JXwn06 or genotype 1 strain GZ11-G1 with the MOI of 0.01, respectively, as described previously [8, 28]. At 36 h post-inoculation, cells were fixed with 80% acetone and treated with 0.1% Triton X-100 (Sigma–Aldrich) at room temperature for 10 min, followed by blocking with 2% BSA for 30 min, and then the cells were incubated with screened out nsp11 specific antibody or anti-PRRSV N monoclonal antibody SDOW-17 at 37°C for 1 h. After washing three times with PBS, the cells were then incubated with fluorescein isothiocyanate (FITC)-conjugated goat anti-mouse IgG (Zhongshan Jinqiao Bio, Inc) at 37°C for 1 h. Followed with washing three times with PBS, the stained cells were visualized with a fluorescence microscope fitted with a camera (Nikon).

### Accurate position of epitope identification

To generally map the epitope position to anti-nsp11 mAb 3F9, four overlapped fragments covered the nsp11_40-223aa_ were cloned into the expressing vector PEGFP-N1, respectively, then the recombinant proteins were expressed in transfected HEK 293 cells. The truncated proteins were verified the reactivity with anti-nsp11 mAb 3F9 by western blotting. On the basis of WB results, the mAb 3F9 recognized fragment was further divided into 9 smaller overlapped fragments to further repeat the mapping. And at the final round, fragments with only 6aa or 7 aa were tested in WB to identify the accurate position of epitope. The region of each fragments and PCR primers used for amplification were listed in Table 1.

### Western blotting

The western blotting (WB) was performed as following, the transfected HEK 293 cells, MARC-145 cells inoculated with JXwn06 or GZ11-G1 and mock infecting cells were subjected to SDS-PAGE, and then electrotransferred to PVDF membrane (Millipore). After blocking with 5% skim milk at 37°C for 2h, the membrane was incubated with anti-nsp11 mAb 3F9 (diluted 1:500 in blocking buffer) at 37°C for 2h, then washed three times with PBST. Finally, the membrane was probed with a 1: 5, 000 diluted HRP-conjugated goat anti-mouse IgG (Sigma–Aldrich) at 37°C for 1 h. The reactivity was visualized with electrochemiluminescence (ECL) reagents (Vigorous bio).

### Statistical analysis

A student t test was used to analyze the significant difference of OD value between positive and negative serum samples, or between control and mAb 3F9 in indirect ELISA. Differences were analyzed by GraphPad Prism software (version 5.0) and were considered statistically significant at a *P* value of <0.05.

### Bioinformatics analysis

To investigate if the identified epitope was conserved among different genotypes of PRRSV, the sequence of epitope region among different reference strains were compared by using Megalign in DNASTAR software. As well, the spatial distribution of identified epitope was analyzed by mapping the location on the 3D structure model of PRRSV nsp11 [21, 31] by using PyMOL software.

## Results

### Expression and purification of His-tagged nsp11 recombinant protein

**Fig 1:**
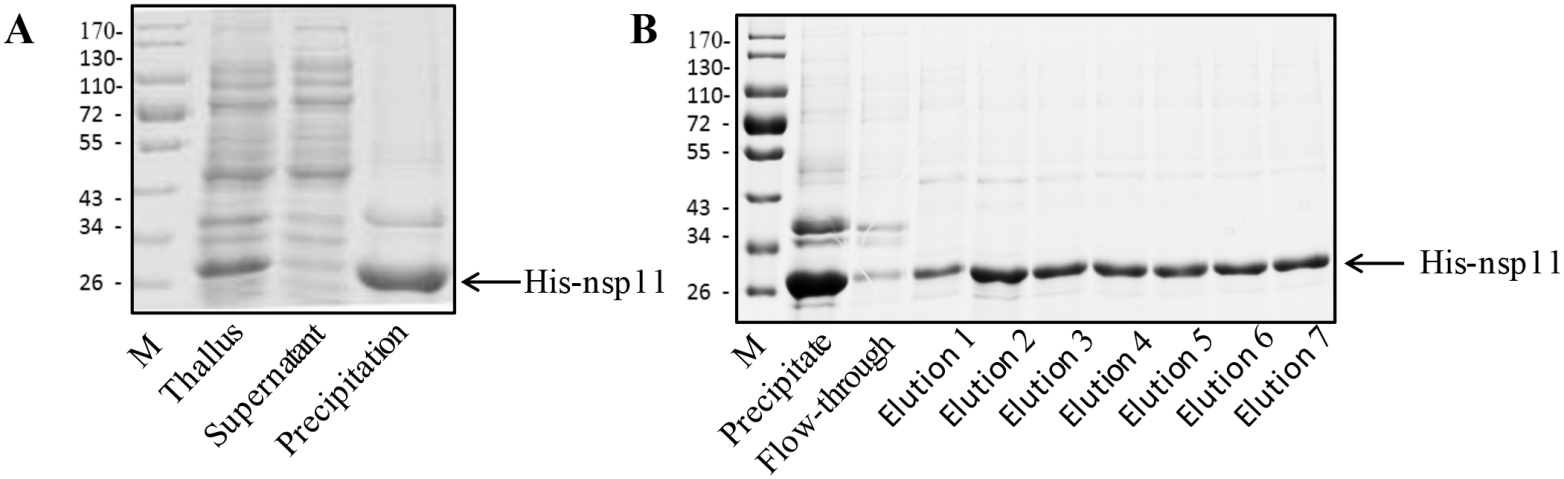
Expression and purification of His-tagged nsp11_40-223aa_ protein. (A) After culture and IPTG induction, the plasmid pET28a-nsp11 transformed *E* coli cells were collected. The thallus, as well as supernatant and precipitation parts post lysing, were submitted to SDS-PAGE for detecting the nsp11 expression. (B) The purity and concentration of purified nsp11_40-223aa_ protein was analyzed in SDS-PAGE.

The His-fused recombinant protein His-nsp11_40-223aa_ was successfully expressed in *E coli* BL21 cells with the expected size of 26 kDa (Fig 1A). After ultra-sonication, the expressed protein was detected in precipitation part but not in supernatant, indicating that the His-nsp11_40-223aa_ predominately existed in the inclusion bodies. The His-nsp11_40-223aa_ protein was subsequently purified by Ni-Agarose His Purification Kit and the purified protein showed as a single band in SDS-PAGE with molecular weight of approximately 26 kDa (Fig 1B).

### Identification and characterization of the anti-Nsp11 monoclonal antibody

**Fig 2:**
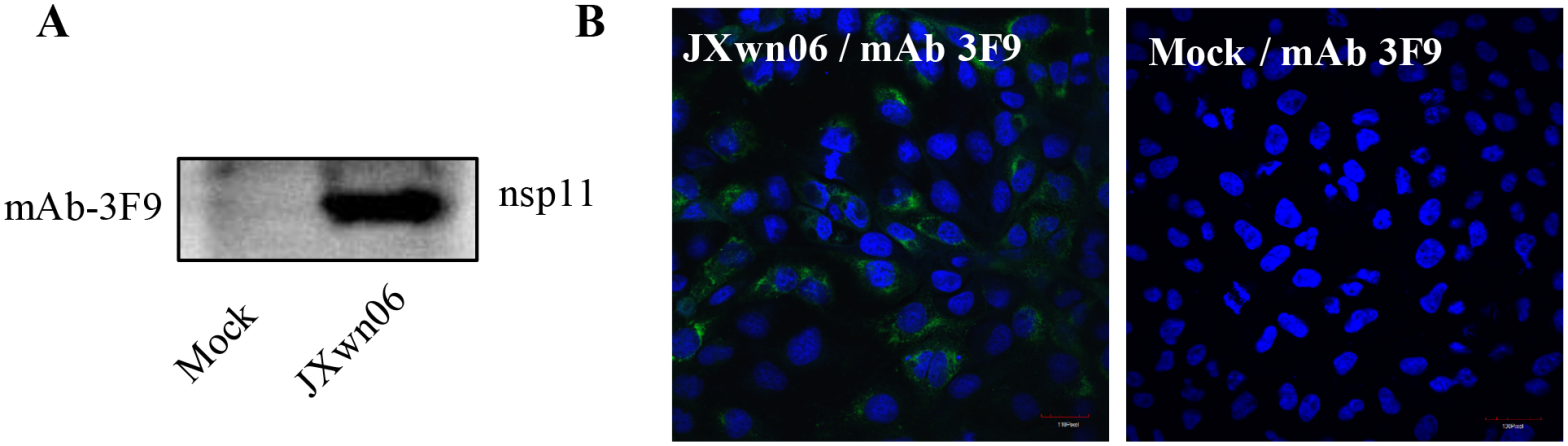
Identification of the anti-nsp11 monoclonal antibody (3F9). The PRRSV JXwn06 infected MARC-145 cells and mock cells were submitted for WB analysis (A) and IFA (B) by using mAb 3F9 as primary antibody.

The purified His-nsp11_40-223aa_ protein was used to immunize BALB/c mice to generate hybridoma cells secreting antibodies specifically against the PRRSV nsp11. An hybridoma cells clone, named as 3F9, was identified by testing its supernatants using PRRSV nsp11-specific indirect ELISA. And following isotyping results showed that mAb 3F9 belongs to subclass IgG1/κ-type. To further confirm the specificity of the mAb 3F9, the PRRSV JXwn06 infected MARC-145 cells were analyzed by WB and IFA using the mAb 3F9 as the primary antibodies. The results of WB revealed that the mAb 3F9 could specifically react with Nsp11 protein expressed in PRRSV infected cells but not with mock infected sample (Fig 2A). As well, the specific positive signal of green fluorescent could be also observed in the infected cells (Fig 2B).

### Precise definition of the novel epitope recognized by mAb 3F9

**Fig 3:**
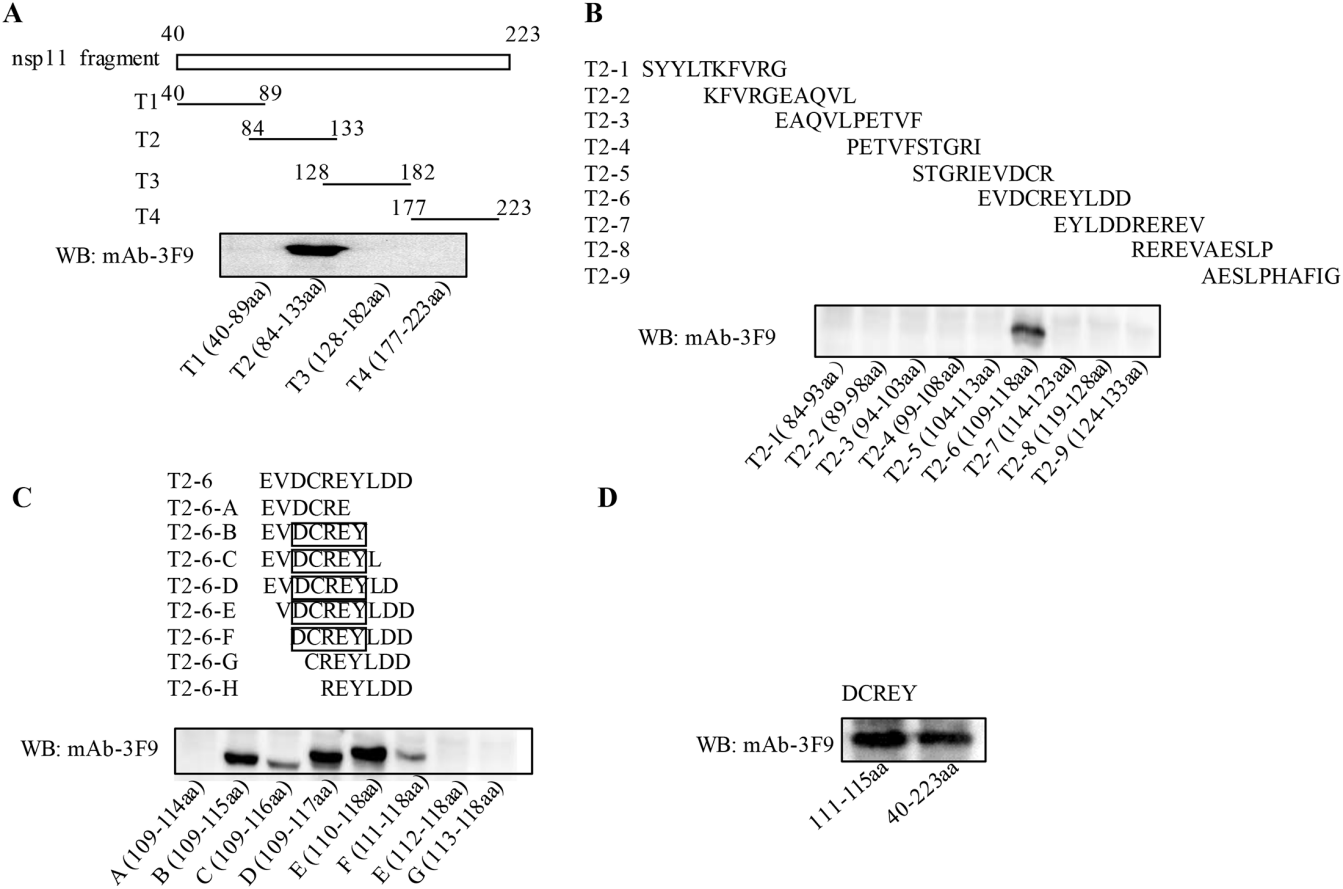
Identifying B-cell epitope of 3F9. (A) Four overlapped fragments covered the nsp11_40-223aa_ were expressed by using vector PEGFP-N1, and then the mAb 3F9 reactivity of truncated nsp11 proteins were verified by WB. (B) The mAb 3F9 reactivity of nine overlapped fragment covered nsp11_84-133aa_ were further tested by WB. (C) Eight overlapped fragments covered the nsp11_109-118aa_ were further tested by WB. (D) The identified core unit was expressed in PEGFP-N1, for testing its mAb 3F9 reactivity by WB, with the nsp11_40-223aa_ as positive control.

To identify the mAb 3F9 recognized B-cell epitope, four overlapped fragments covered the nsp11_40-223aa_ were cloned into the PEGFP-N1, expressed as recombinant proteins, to react with mAb 3F9 in WB (Fig 3A). Subsequently, the mAb 3F9 recognized T2 fragment covered 84-133aa of nsp11 was further divided into nine overlapped shorter fragments to test with mAb 3F9 (Fig 3B). The screened out fragment T2-6, covered 109-118aa of nsp11, was finally divided into eight fragments with at least 6 amino acid overlapped among neighbors, to react with mAb 3F9. As the Fig 3C shown, all fragments from T2-6-B to T2-6-F could be recognized by mAb 3F9 respectively, but the rest T2-6-A, G and H could not, which indicates that the “DCREY” covered 111-115aa of nsp11 is the minimal core unit of epitope recognized by mAb 3F9. And the reactivity was further confirmed by expressing the exact core unit ^111^DCREY^115^ to bind with mAb 3F9, which show the same reactivity as nsp11_40-223aa_ did (Fig 3D).

### Reactivity of the novel epitope with pig serum samples

**Fig 4:**
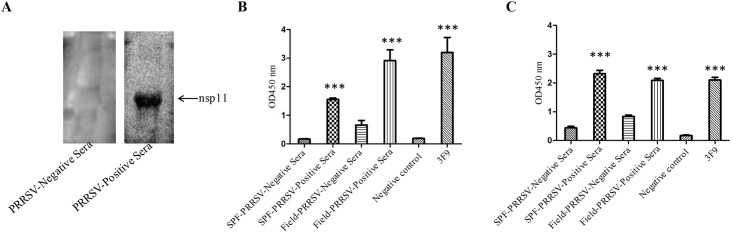
Serological test for PRRSV nsp11 and the identified ^111^DCREY^115^ epitope. (A) The nsp11 specific antibody in anti-PRRSV sera were detected by reacting with nsp11_40-223aa_ protein in WB test. (B) The nsp11 specific antibody in anti-PRRSV sera were detected by nsp11_40-223aa_ coated indirect ELISA. (C)The ^111^DCREY^115^ epitope specific antibody in anti-PRRSV sera were detected by peptide coated indirect ELISA. Asterisk indicates significant differences between PRRSV positive sera and negative sera, or between control and mAb 3F9 (* *P<*0.05; ** *P<*0.01; *** *P<*0.001).

In order to characterize immunogenic reactivity of nsp11 and its identified epitope ^111^DCREY^115^, during PRRSV infection, the expressed His-nsp11_40-223aa_ was submitted to test the reactivity with anti-PRRSV sera in WB, which were collected from previous inoculation study or from field, and identified by IDEXX HerdChek PRRS X3 ELISA kit. As the Fig 4A shown, the expressed His-nsp11_40-223aa_ protein could be recognized by anti-PRRSV sera, but it did not react with PRRSV negative sera. Subsequently, ELISAs were performed using either His-nsp11_40-223aa_ or synthetic peptide ^111^DCREY^115^ as coating antigens, and the anti-PRRSV sera from inoculation study or pig farms were further tested, with the mAb 3F9 as partial control. The OD_450_ value of PRRSV positive sera was significant higher than that of PRRSV negative sera (*P*<0.001), in both His-nsp11_40-223aa_ and peptide coated ELISA test. The results of WB and ELISA indicate that PRRSV nsp11 could induce the humoral immunity responds in pig during PRRSV infection and the epitope ^111^DCREY^115^ could be recognized by anti-PRRSV sera as well.

### Conservation of identified epitope between different PRRSV genotypes

**Fig 5:**
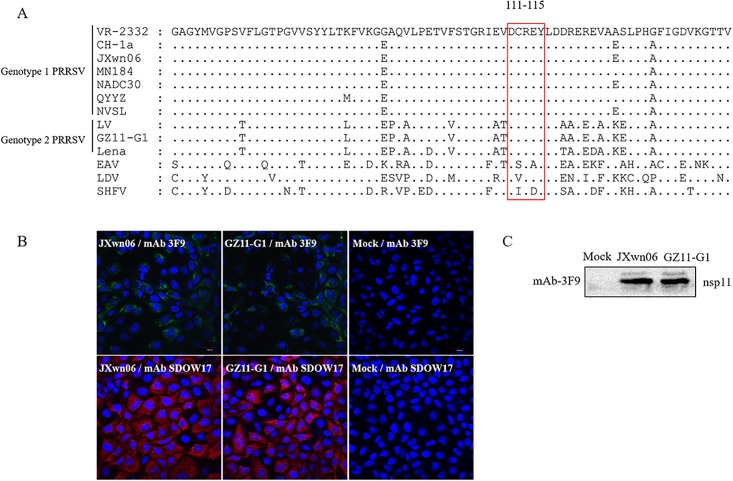
Confirm the conservation of identified ^111^DCREY^115^ epitope in both genotype PRRSV. (A) Multiple sequence alignment of nsp11 epitope region. The identified epitope was labeled with box. (B and C) The PRRSV JXwn06 (genotype 1) or GZ11-G1 (genotype 2) inoculated MARC-145 cells were detected in IFA (B) and WB (C), by using nsp11 specific mAb 3F9 and N protein specific mAb SDOW17(for IFA only) as primary antibody.

The sequence of epitope region among different PRRSV reference strains from both genotypes, together with EAV, LDV and SHFV, were aligned by using Megalign in DNASTAR software, and the results show that the identified nsp11 epitope ^111^DCREY^115^ was highly conserved between the two genotypes (Fig 5A), but not conserved among Arterivirus. JXwn06 and GZ11-G1 represented each genotypes were used to inoculate MARC-145 cells, followed by IFA and WB test, the result confirmed that both virus can be recognized by mAb 3F9 (Fig 5B and 5C), indicating the conservation of this epitope between the two genotype PRRSV.

### Spatial distribution of the novel epitope

**Fig 6:**
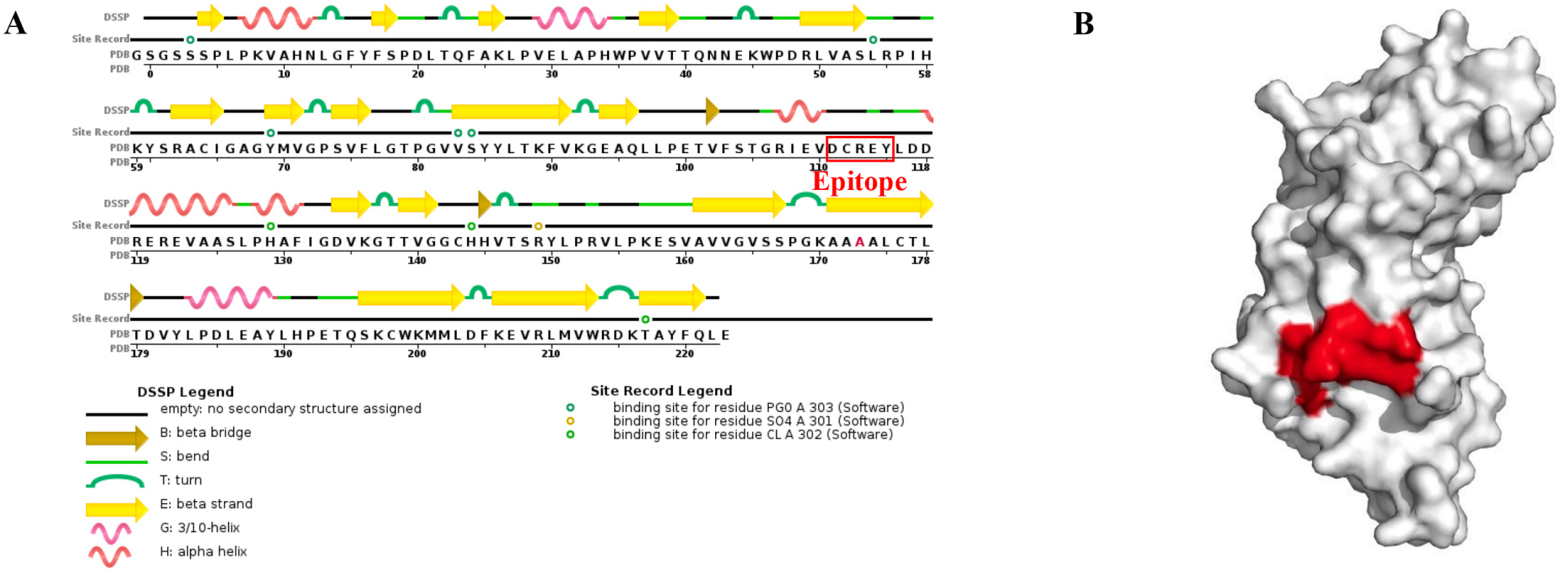
Localization and spatial distribution of the identified ^111^DCREY^115^ epitope. (A) The identified epitope was labeled in the sequence chain view picture of PRRSV nsp11, obtained from the Protein Data Bank (PDB) with the ID 5EYI. (B) Spatial distribution of the identified epitope on the nsp11.

To localize the identified epitope ^111^DCREY^115^, a sequence chain view picture of PRRSV nsp11 was obtained from the Protein Data Bank (PDB) with the ID 5EYI (Fig 6A), and the identified epitope is found to locate at the region without secondary structure assigned or bend region, which is responding to its characterization as liner epitope. And the spatial distribution was also analyzed on the 3D structure model of PRRSV nsp11 by using PyMOL software. The structural visualization showed that the identified epitope recognized by 3F9 is exposed on the surface of the nsp11 structure (Fig 6B).

## Discussion

30 years ago, PRRS is initially named as “mystery disease”, due to unknowing its pathogen at the early outbreak [32]. Considering many mechanisms of its pathogenesis, replication and immune regulation are unclear, even now, the PRRSV is still a mystery virus. The nonstructural proteins play amounts of essential biological roles in viral replication and/ or host immune response regulation by virus-host interaction. Among them, PRRSV nsp11 with the NendoU domain presents its necessary for viral genome replication and contributes to inhibiting host innate immunity through its endoribonuclease and deubiquitination activity. However, the immunogenicity and immune structure of PRRSV nsp11 has not been clear yet. In this study, the expressed His-tagged nsp11 recombinant protein was used to immunize BALB/c mice for mAb preparation and the mAb 3F9 belonging to a subclass IgG1/κ-type was successfully identified. Further, the mAb 3F9 specifically binding epitope with the exact core unit ^111^DCREY^115^ was also successfully identified.

In previous research, many epitopes in different nonstructural proteins of PRRSV, including nsp2, nsp10 and nsp12 were identified and reported [30, 33, 34]. Depending on the conservation or variation of these epitopes among different kind of PRRSV strains, their specific mAbs can be used as tools for differentiating the two genotype strains or HP-PRRSV from classic PRRSV strains [33, 34]. Here, the core unit ^111^DCREY^115^ was found to be conserved in both genotypes of PRRSV, but not in Arterivirus. Only three residues “REY” at 113-115aa were conserved in PRRSV, LDV and HSFV [21]. However, due to lacking other Arterivirus in our lab, the cross-activity of 3F9 mAb with other members of Arterivirus was not carried out this time.

The core unit “DCREY” was located at 111-115aa of PRRSV nsp11, close to the start region of NendoU C-terminal catalytic domain (R107 to E223). Among this core unit, the residue C112 was regarded to interact with residue R153 from another subunit, which might be important for the hydrophilic and hydrophobic interactions between monomers and supporting the formation of stable dimers [21]. So it is very interesting, weather this antibody could interrupt the function of PRRSV nsp11 by interrupting the dimers formation, if it has been overexpressed in PRRSV infected cells. Further, the 3D structural visualization shown that this epitope presents at the surface of nsp11 [21]. This characterization makes this epitope easy to explore and induce host immune response in infected animals, which was confirmed by serological test using PRRSV positive sera. Together with its conversation between two genotype virus, this nsp11 B-cell epitope could be a good target for both serological and pathogen detection.

In summary, a novel mAb 3F9 against the nsp11 of PRRSV was generated and the core motif ^111^DCREY^115^ of its specific B-cell epitope, which is conserved in both genotypes of PRRSV, was successfully identified. Our finding does not only provide an mAb tool to further investigate the function of nsp11 such as replicase localization or viral-host protein interaction, it also indicates great diagnostic potential for this epitope.
